# Supplementary material for: Detection and quantification of key dental pathogens through wastewater monitoring
Source: PLoS One. 2025 Nov 6;20(11):e0328420. doi: 10.1371/journal.pone.0328420 (PMC12591483; doi:10.1371/journal.pone.0328420)
Supplement: S1 Table — (DOCX) [file pone.0328420.s001.docx]

**Table S1:** Primers and probes for each of the targets used throughout the study.

| Organism | Type | Sequence | Citation |
| --- | --- | --- | --- |
| *S. mutans* | Forward | GCCTACAGCTCAGAGATGCTATTCT | [1] |
|  | Reverse | GCCATACACCACTCATGAATTGA |  |
|  | Probe | FAM- TGGAAATGACGGTCGCCGTTATGAA-BHQ1-3 |  |
| *P. gingivalis* | Forward | GCGCTCAACGTTCAGCC | [2] |
|  | Reverse | CACGAATTCCGCCTGC |  |
|  | Probe | HEX- CACTGAACTCAAGCCCGGCAGTTTCAA-BHQ1-3 |  |

**Citations**

1. Yoshida, A., et al., *Development of a 5′ Nuclease-Based Real-Time PCR Assay for Quantitative Detection of Cariogenic Dental Pathogens Streptococcus mutans and Streptococcus sobrinus.* Journal of Clinical Microbiology, 2003. **41**(9): p. 4438-4441.

2. Boutaga, K., et al., *Comparison of Real-Time PCR and Culture for Detection of Porphyromonas gingivalis in Subgingival Plaque Samples.* Journal of Clinical Microbiology, 2003. **41**(11): p. 4950-4954.
