# Supplementary material for: Detection and quantification of key dental pathogens through wastewater monitoring
Source: PLoS One. 2025 Nov 6;20(11):e0328420. doi: 10.1371/journal.pone.0328420 (PMC12591483; doi:10.1371/journal.pone.0328420)
Supplement: S2 Fig — (DOCX) [file pone.0328420.s003.docx]

**Figure S2.** Comparison in bacterial concentrations between WWTP’s. Top two graphs show distribution of the concentration of *S.mutans* (left) and *P. gingivalis* (right) in wastewater from DR and BP in terms of copies/L. Samples in which bacteria were not detected were removed. Bottom graphs show wastewater concentrations of *S.mutans* (left) and *P. gingivalis* (right) in DR and BP during the winter. The solid line represents the 3-sample smoothed and trimmed average.
