# Supplementary material for: Detection and quantification of key dental pathogens through wastewater monitoring
Source: PLoS One. 2025 Nov 6;20(11):e0328420. doi: 10.1371/journal.pone.0328420 (PMC12591483; doi:10.1371/journal.pone.0328420)
Supplement: S3 Fig — (DOCX) [file pone.0328420.s004.docx]

**Figure S3.** Comparison in microbial concentrations between for year 1 and year 2 using Pearson’s correlation 2.
