# Supplementary material for: Detection and quantification of key dental pathogens through wastewater monitoring
Source: PLoS One. 2025 Nov 6;20(11):e0328420. doi: 10.1371/journal.pone.0328420 (PMC12591483; doi:10.1371/journal.pone.0328420)
Supplement: Attachment — Submitted filename: Response to reviewers.docx [file pone.0328420.s005.docx]

**Response to Reviewer 1**

The authors appreciate the reviewer for their time and consideration when reviewing the submitted manuscript.

All changes made in response to the comments have been incorporated into the revised manuscript and are highlighted for clarity.

Original reviewer comments are noted below in italics, followed by author responses

**Major comments:**

*It seems three different studies, i.e., (i) detection of the dental pathogens in wastewater, (ii) reduction of these pathogens in a WWTP, and (iii) decay of these pathogens in wastewater, are mixed without proper coherence.*

**1**. *If the study aims to evaluate potential applicability of WBE for the dental pathogens, more in-depth study on its accuracy and validation is crucial. For example, evaluation of recovery efficiency in the detection methodology using samples with known concentration, comparison with the expected concentration which is calculated from shed quantity of the target pathogens in sputum and feces from the past clinical studies or estimated infected population from such data, comparison among different WWTPs which serve the area with different dental conditions.*

**Response:**

The authors appreciate this insightful comment regarding the importance of assessing the applicability of WBE for dental pathogens. While we were not able to assess the shedding dynamics of target pathogens and estimate the infected population in the current area due to the gap in current research on these pathogens, we did perform sequencing analysis on the samples with the highest concentration to validate the presence of target pathogens and compare them to previous sequences. We also performed experiments on the different WWTPs in the area but could not determine differences between dental conditions in the different locations because routine monitoring for these pathogens is not done. However, our results show that the concentrations of target pathogens between the two areas are similar. We fully agree that establishing recovery efficiency in future studies is necessary and have included this in the limitations section of our manuscript.

Line 205-217: Amplicon sequencing was done for the detection of *S. mutans* and *P. gingivalis* using primers developed in this study. For *S. mutans*, the targeted gene was glucosyltransferase-I (gtfB) using the forward and reverse primers ATCATTACGTCTGTCCGCTATG and CATCGGCTGTCCCGTATTT, respectively. For *P. gingivalis*, the targeted gene was 16SrRNA and the forward and reverse primers used were CCCGTTGAAAGACGGACTAAA and CTTCAGTGTCAGTCGCAGTATG, respectively. The amplification for s. mutans resulted in a 691-nt product. The amplification of p. gingivalis produced a 606-nt product. For the qPCR, each reaction contained 10 μL of HotStarTaq® Plus Master Mix (Qiagen, Cat. No. 203645; Hilden, Germany), 2 μL of 1× HST buffer, and 5 μL of template DNA. The thermal cycling program began with a 15-minute activation at 95 °C, followed by 50 PCR cycles with denaturation at 95 °C for 5 seconds, annealing at 55 °C for 30 seconds, and extension at 72 °C for 1 minute.

Line 508-517: To strengthen and validate the applicability of WBE for tracking dental pathogen prevalence, the recovery efficiency of the detection methodology should be assessed using spiked samples with known concentrations of target pathogens. Due to the limited availability of clinical data for dental pathogens, comparisons between observed concentrations in wastewater and expected levels based on pathogen shedding rates from sputum or feces were not done. Still, they would help to improve the estimation of infection prevalence in communities. Another comparison that future studies may consider is to include WWTPs with different dental conditions, which would provide more insight into the relationship between detection and oral health.

SI Line 50-57:

**Figure S2.** Comparison in bacterial concentrations between WWTP’s. Top two graphs show distribution of the concentration of *S.mutans* (left) and *P. gingivalis* (right) in wastewater from DR and BP in terms of copies/L. Samples in which bacteria were not detected were removed. Bottom graphs show wastewater concentrations of *S.mutans* (left) and *P. gingivalis* (right) in DR and BP during the winter. The solid line represents the 3-sample smoothed and trimmed average.

**2.** *What are the public health implications of their reduction in a WWTP? If the study wants to evaluate reduction performance, a discussion on their risk is inevitable. Currently, how much of the risk is posed by WWTP effluent through exposure to the target pathogens in environmental waters receiving the WWTP effluent? And how much is the required concentration or reduction efficiency to meet the acceptable risk?*

**Response:**

The authors agree that the public health implications of these dental pathogens is important to consider and appreciate the reviewer mentioning this alongside transmission. The transmission of these dental pathogens has typically been vertical transmission through kissing or improper oral hygiene and with recent studies showing the possibility of horizontal transfer, this creates the possibility of introduction of these pathogens from wastewater into the oral cavity. The risk that these dental pathogens pose in wastewater effluent is a limitation that we have not included in our study before, but we now realize the importance of including it. These revisions have been included in the manuscript.

Line 452-461: It has been proven in past studies that the possibility of horizontal transmission of dental pathogens, like *S. mutans*, is possible, whether this be through kissing, shared drinks, or other forms of contact with infectious fluids (Baca et al., 2012; Momeni et al., 2016). Although the risk of transmission of dental pathogens from wastewater effluent to the oral cavity has not been fully studied, this risk can be assessed similarly to that of enteric viruses entering via the fecal oral route. When considering an acceptable or required reduction efficiency in the wastewater treatment process, future studies should examine the relationship between bacterial load and the risk of contracting an infection, as well as the infectivity of the target pathogens identified.

**3.** *L.211: Calculation of normalization should be clarified in the method section. It is weird that the chart still indicated as gc/L after normalization. To normalize with the flow rate, daily total pathogen load as gc/d or gc/d/capita should be used.*

**Response:**

The authors agree that more clarity should be provided in the method section on the normalization process. Concentrations were multiplied by a flow rate and population (through crAssphage) correction factor that was calculated by determining the average flow rate across the year, then dividing the monthly flow rate by the annual average. These details are placed in the text below.

Line 196-200: The flow rate and population (population normalization done through crAssphage) correction factor were calculated by determining the average flow rate or crassphage concentrations across the year then dividing the monthly values by the annual average value. Each sample concentration was then multiplied by both the flow rate and population correction factors.

**4.** *I cannot get the purpose of the decay experiment for the main objective of this study. If it aims to evaluate the impact of sample storage, it should be discussed with methodology evaluation as commented above.*

**Response:**

The authors appreciate this insight into the confusion and we agree that, as presented, the decay experiment does not align well with the current study. This experiment has been removed from the manuscript to improve focus and clarity.

**Minor comments:**
**1**. *Wastewater is suggested to include pathogens shed from sputum by several literature. I suggest to review them and include them in the introduction.*

**Response:**

The reviewer is correct that wastewater includes sputum, and the manuscript has not included this information, as well as appropriate literature cited.

Line 69-71: It has also been determined in previous studies that wastewater includes pathogens shed by sputum as well, which could be a strong access route for these dental pathogens (Lowry et al., 2023)

**2**. *Figure 1 is more appropriate in the supplement. Instead, crAssphage data in Figure S1 should be shown in the main text.*

**Response:**

The authors appreciate this insight and have now included the crAssphage data into the manuscript and the figure originally in Figure 1 is now placed in the supplementary.

**Figure 1.** monthly average concentrations of crAssphage in Bloomington wastewater over the two-year duration. The bars are standard deviation for four samples collected each month (n=4-5).

**Response to Reviewer 2**

The authors would like to thank the reviewer for their time and consideration in reviewing our paper, as well as the kind words and support.

All changes made in response to the comments have been incorporated into the revised manuscript and are highlighted for clarity.

Original reviewer comments are noted below in italics, followed by author responses

**Reviewer 2 Specific Comments:**

**1.** *Line 33. This sentence could be interpreted as suggesting that 100 samples were taken every week. Please check and correct if that’s not the case.*

**Response:**

The authors understand how the wording may be confusing and have since revised the statement for clarity.

Line 33-35: Between June 2023 and May 2025, a weekly sample of untreated wastewater was collected, resulting in a total of 100 samples collected over the timespan.

**2.** *Lines 41-42, 83-85 and elsewhere. Nothing in this paper or the analysis presented can speak directly to the ability of these pathogens to be transmitted by water, either drinking water or recreational exposure. This should be removed as an unsupported claim.*

**Response:**

The authors appreciate this insight and have now provided information about how previous studies have shown the possibility of horizontal transfer through individuals through kissing and improper oral hygiene. This possibility opens up the potential for it to be transmitted through water, including wastewater, but future studies should test the infectivity of these particles in wastewater as well. This information has now been included in the manuscript and appropriately cited.

Line 451-460: It has been proven in past studies that the possibility of horizontal transmission of dental pathogens, like *S. mutans*, is possible, whether this be through kissing, shared drinks, or other forms of contact with infectious fluids (Baca et al., 2012; Momeni et al., 2016). Although the risk of transmission of dental pathogens from wastewater effluent to the oral cavity has not been fully studied, this risk can be assessed similarly to that of enteric viruses entering via the fecal oral route. When considering an acceptable or required reduction efficiency in the wastewater treatment process, future studies should examine the relationship between bacterial load and the risk of contracting an infection, as well as the infectivity of the target pathogens identified.

**3.** *Introduction, lines 96-98, and elsewhere. The authors make a case that better surveillance of these pathogens could be used in targeting interventions. What sort of interventions would be appropriate? Either in the Introduction or Discussion, the authors need to provide a clear “use case” for oral pathogen monitoring by wastewater. The case should include a description of how surveillance for these pathogens is done currently and why WBE is a superior or at minimum a complementary method that can result in actionable information. Otherwise, the findings are potentially interesting but not that valuable from a public health monitoring perspective.*

**Response:**

The authors understand the reviewers’ concern and have added more information on targeting interventions and a clearer “use case” of oral pathogen monitoring in wastewater. The following text has been added to the revised manuscript.

Line 75-82: Current approaches to monitoring oral health at the population level rely heavily on dental claims data and self-reported surveys. However, these methods have notable limitations. Claims data often exclude individuals without access to dental insurance, while self-reported surveys are subject to recall and reporting biases and are typically conducted infrequently (Okunseri et al., 2023). As a result, these approaches may fail to capture timely, representative data, especially among vulnerable populations who already face barriers to dental care and experience higher burdens of oral disease.

Line 105-116: These insights could be used to inform or evaluate public health interventions such as community water fluoridation programs, school-based dental screenings, or targeted outreach in high-incidence areas(Melbye and Armfield, 2013). Importantly, integrating WBE into oral health surveillance frameworks could help address long-standing inequities by identifying underserved populations that might otherwise be missed using conventional data sources. This capability supports a more proactive and inclusive approach to public oral health planning and resource allocation. Wastewater-based epidemiology (WBE) represents a complementary and potentially more equitable surveillance tool. Because WBE captures pooled biological signals from entire communities regardless of socioeconomic or insurance status, it has the capacity to provide anonymized, near real-time insights into population-level trends in oral pathogen circulation.

**Response to Reviewer 3**

The authors appreciate the reviewer for their time and consideration when reviewing the submitted manuscript.

All changes made in response to the comments have been incorporated into the revised manuscript and are highlighted for clarity.

Original reviewer comments are noted below in italics, followed by author responses

**Specific Comments:**

**1.** *Line 70: How strong a correlation between wastewater bacterial levels and oral disease can be established. Please explain more in details.*

**Response:**

The aforementioned statement in the manuscript was unclear and confusing, and the authors appreciate this insight. The sentence has now been revised and improved for clarity.

Line 71-75: If a strong correlation between wastewater bacterial levels and oral disease prevalence can be established, then this approach could serve as an early warning system for emerging trends in oral health conditions, prompting timely public health interventions (Kasprzyk-Hordern et al., 2022; Mao et al., 2020).

**2.** *Line 93: What do you mean by a month-long decay experiment? Why do authors consider a month-long decay experiment? Is there any specific reason for this?*

**Response:**

As mentioned in the previous responses, the decay portion of the experiment has since been removed from the manuscript, as it does not align well with the other experiments.

**3.** *Line 108: Please provide the total catchment population if available for each WWTP*.

**Response:**

The total catchment population for each wastewater treatment plant was obtained and has now been included in the manuscript.

Line 121-123: Blucher Poole has a flow of more than 4.5 million gallons per day and caters to the northern part of Monroe County with an estimated total catchment population of 25,000 individuals

Line 128-131: Dillman Road is located on the south side of Bloomington, Indiana, and has a peak hydraulic capacity at 30 million gallons daily with an estimated total catchment population of 55,000 individuals (Bloomington, 2023).

**4.** *Line 136: The authors mentioned that the flasks were covered with parafilm and left in the dark room. What about the temperature? Did the authors consider the temperature variation during the study period? If no, why? Temperature could affect the decay of the pathogens.*

**Response:**

The authors understand the concern with the effect of temperature on decay and have considered it. However, the decay portion of the experiment has since been removed from the manuscript.

**5.** *Line 138: Is there any reason for selecting the specific time/day for monitoring the decay rate?*

**Response:**

The authors did not have any reason for selecting the specific time/day for decay monitoring, but understand how this information would be important to consider. The decay portion of the experiment has now been removed from the manuscript as per previous reviews.

**6.** *Line 143: Why were all samples acidified to a PH of 3.5? Please explain it in detail, also, how it was done.*

**Response:**

All samples were acidified to a pH of 3.5 to ensure the recovery of the target pathogens and enhance binding. The authors appreciate this question and have added supplemental information to the manuscript. This information includes how to change the pH

Line 152-153: All samples were acidified to a pH of 3.5 by the addition of 20 µL of Hydrochloric Acid to increase the electrostatic attraction of the bacteria inside the sample to the negatively charged filters.

**7.** *Line 151: Allprep should be replaced with AllPrep.*

**Response:**

The authors appreciate this attention to detail and have since corrected the mistake in the manuscript.

Line 161: AllPrep

**8.** *Line 167: Samples were tested in duplicate; how did the authors treat the one-well positive samples? Did the author consider LOD and LOQ values?*

**Response:**

The author appreciates the reviewer’s comment and understands how the previous manuscript description may have been confusing. The manuscript has now been edited for clarity.

Line 184-187: The lowest detection limit was estimated for each PCR assay using the value of one positive well partition from DNA controls, which resulted in a calculated 380 copies/L for both *S. mutans* and *P. gingivalis*(Greaves et al., 2020). All non-detection samples were reported as the limit of detection.

**9.** *Line 211: Authors normalize pathogen concentrations using flow rate, which helps adjust for basic dilution. The authors also mentioned the stable detection of crAssphage throughout the study period. However, flow rate can vary due to other environmental factors like rainfall, industrial inputs, or groundwater infiltration, which may not reflect true changes in the human population. Did the authors evaluate how sensitive their results are to these factors, or consider whether crAssphage normalization would provide a more robust population indicator?*

**Response:**

The authors recognize the importance of other factors, such as environmental factors, which can impact the quantification values and alter the flow rate. The sewer system from which we obtained samples was not a combined stormflow sewer system; however, there could be other factors that have the potential to impact the data. The authors have considered this comment and have added this to the limitations section.

Line 519-523: While the sewer system is a separate sanitary system and thus less prone to storm-related flow variability, minor contributions from infiltration or undocumented inputs cannot be entirely ruled out. Future studies incorporating flow composition analysis or additional chemical tracers may help further refine normalization approaches.
