# Supplementary material for: Detection and quantification of key dental pathogens through wastewater monitoring
Source: PLoS One. 2025 Nov 6;20(11):e0328420. doi: 10.1371/journal.pone.0328420 (PMC12591483; doi:10.1371/journal.pone.0328420)
Supplement: Attachment — Submitted filename: Responsetoreviewers round 2.docx [file pone.0328420.s006.docx]

**Response to Reviewer 1**

All changes made in response to the comments have been incorporated into the revised manuscript and are highlighted for clarity.

Original reviewer comments are noted below in italics, followed by author responses and changes in the manuscript.

**Major Comment:**

**Comment:**
1. *The conclusion "this study demonstrated the feasibility of using WBE for oral pathogen surveillance in a community" is not proven in this study. Though detection of dental pathogens is meaningful, that does not mean its utility in WBE. In order to demonstrate its utility, its quantitative change should be verified with clinical or relevant data. The conclusion should be written more carefully by clearly distinguishing what is robust findings supported by evidence from what is merely speculated.*

**Response:**

We thank the reviewer for this thoughtful comment and agree that our study does not establish the full feasibility or utility of wastewater-based epidemiology (WBE) for oral pathogen surveillance. As corresponding clinical data were not available, the findings cannot be directly validated against oral disease prevalence. In response, we have revised the conclusion to clarify that this study demonstrates the detection of oral pathogen genetic material in wastewater and suggests that WBE may serve as a complementary tool when used alongside clinical and epidemiological data, where available. The revised conclusion reads as follows:

Line 544-557: In conclusion, this study demonstrated the detection of oral pathogen genetic material in community wastewater, indicating the potential feasibility of using wastewater-based epidemiology (WBE) for oral pathogen surveillance. The findings also highlight the need for improved public health surveillance of dental and periodontal infections, as the absence of such data limits the ability to validate wastewater observations against clinical trends. Furthermore, while this study cannot confirm an environmental transmission pathway for *S. mutans*, it underscores the importance of future research evaluating pathogen viability and possible exposure routes through environmental water. Overall, this research has the potential to provide critical insights into oral health trends, improve disease surveillance, and enhance public health strategies. By leveraging wastewater analysis alongside clinical and epidemiological data, where available, we can move toward a more proactive and non-invasive approach to oral disease monitoring, ultimately contributing to better health outcomes and reducing the global burden of dental and periodontal diseases.

**Comment:**
2. The conclusion "our study showed a potential environmental transmission pathway for S. mutans through water" is not proven in this study. Thie study only demonstrated the presence of genetic materials of the dental pathogens in WWTP effluent. We cannot prove the environmental transmission unless the followings are not proven (either in this study or in any literature): (1) the presence of not only genetic materials but also infectious viable pathogens, (2) possible infection by ingestion of water (not by mouth-to-mouth contact).

**Response:**

*We thank the reviewer for this insightful comment and fully agree that the current study does not demonstrate an environmental transmission pathway for S. mutans. Our data show the presence of genetic material in wastewater effluent but do not confirm pathogen viability or infectivity. We have revised the manuscript to clarify this limitation and to avoid overstating the conclusion. The text now emphasizes that further studies are required to assess the infectivity of wastewater samples and to evaluate whether ingestion of contaminated water could serve as a potential route of transmission. The revised statement can be found in the limitations section.*

Line 537-541: Because the current study detected only genetic material, it cannot confirm an environmental transmission pathway for *S. mutans* or *P. gingivalis,* only speculate the possibility. Future work should specifically evaluate the infectivity of wastewater and environmental samples, as well as investigate whether ingestion of contaminated water could plausibly lead to infection.

**Comment:**
*3. L.361-L.366: I wonder if these studies provide quantity of pathogen shedding from saliva and feces. If so, authors can calculate the expected population from the bacterial concentration in wastewater by assuming typical volume/mass of saliva and feces discharged daily from a person. Then, I wonder if the authors can discuss its plausibility by comparing with typical proportion of infected population from past clinical studies.*

**Response:**

We appreciate this thoughtful suggestion. While the referenced studies identified the presence of the pathogen in saliva and feces, they did not provide quantitative shedding rates (e.g., genome copies per liter or per gram). Due to the lack of reported shedding concentrations, it was not possible to perform an informed back-calculation to estimate the infected population or compare it with infection prevalence from clinical data. Additionally, pathogen-specific dental monitoring has not yet been conducted in Indiana, limiting our ability to validate such estimates with local data. We have clarified this limitation in the revised manuscript

Line 508-514: Additionally, while previous studies have reported the presence of these pathogens in saliva and feces, they generally do not include quantitative shedding data (e.g., genome copies per gram or per milliliter). This lack of quantitative information prevents reliable back-calculation of infected population estimates from wastewater concentrations. Furthermore, pathogen-specific wastewater monitoring has not yet been conducted in Indiana, making it difficult to validate such estimates or assess their plausibility against local infection prevalence data.
